# Hybrid Organic/Inorganic Nano-I-Beam for Structural Nano-mechanics

**DOI:** 10.1038/s41598-019-53588-2

**Published:** 2019-12-04

**Authors:** Salah A. M. Elmoselhy

**Affiliations:** Department of Energy and Environment, School of Engineering and Environmental Sciences, University of Applied Sciences Upper Austria (FH OÖ), Campus Wels, Stelzhamerstr. 23, 4600 Wels, Austria

**Keywords:** Applied physics, Condensed-matter physics, Nanoscale materials, Structural materials, Mechanical engineering

## Abstract

For years Carbon nano-tube has shown merits in industrial applications including high structural strength-to-weight ratio. However, from structural mechanics perspective the tube geometrical cross-section is less favored for providing high structural stiffness and strength. Hybrid Organic/Inorganic Nano-I-Beam is thus introduced for improved Structural Nano-mechanics. It has been found that both Wide Flange Nano-I-Beam and Equal Flange & Web Nano-I-beam provide higher structural stiffness and less induced stress and thus longer service life than Nano-Tube. It has been also found that Wide Flange Nano-I-Beam provides higher structural stiffness and less induced stress and thus longer service life than Equal Flange & Web Nano-I-beam. A thermodynamic model of the growth of nano-tubes accounting for vibrational entropy is presented. The results have cost-effectively potential benefit in applications such as nano-heat engines & sensors.

## Introduction

Carbon nanotubes (CNTs) are allotropes of Carbon. They are made of rolled graphene sheets with a nanostructure that can have a length-to-diameter ratio as great as 1,000,000 resulting in distinguished properties. Carbon is exceptional because of its abundance as the 6th most abundant element in the universe and its ability to bond to many elements in many ways^1^. Worldwide commercial interest in CNTs is reflected in a production capacity that presently exceeds several thousand tons per year, serving several industrial applications such as boat hulls, rechargeable batteries, super-capacitors, actuators, and thin-film electronics^2^. However, CNTs are not yet providing compelling mechanical strength or electrical or thermal conductivities for many applications^2^.

Composite materials with CNT and graphene additives have long been considered as exciting prospects among nanotechnology applications^3^. However, after nearly two decades of work in the area, questions remain about the practical impact of nanotube and graphene composites addressing poor load carrying capacity/transfer^3^. Thus, researchers and industrialists in this field are on the outlook for developing high-strength, low-density, high-conductivity materials that can outperform Carbon nano-tubes^3^.

**Figure 1:**
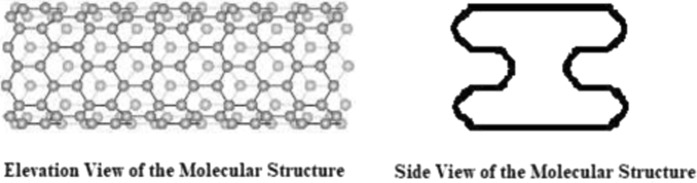
Molecular Form of Carbon Nano I-shaped Like Beam Formed in Zigzag Lattice Vector of Chirality (n, 0).

Addressing the aforementioned limitations, the present work proposes a molecular form of I-shaped like beam, as shown in Fig. 1. The proposed Nano-I-Beam imparts the inherent structural stiffness and inherent structural strength of the I-shaped like beam onto the nano-structure. The molecular form of carbon nano I-shaped like beam formed in zigzag lattice vector of chirality (n, 0), is shown in Fig. 1.

The present research idea fits: (i) One-dimensional (1-D) nano-scale applications such as nano-tubes and nano-rods where electron confinement occurs in 2-D while electron delocalization takes place along the long axis, (ii) two-dimensional (2-D) nano-scale applications such as nano-films and nano-coating, (iii) three-dimensional (3-D) nano-scale applications such as matrix-reinforced nano-composites and multi-nano-layers. The synthesis and growth of nano-I-beam are first investigated herein.

## Synthesis and Growth of Nano-I-Beams

**Figure 2:**
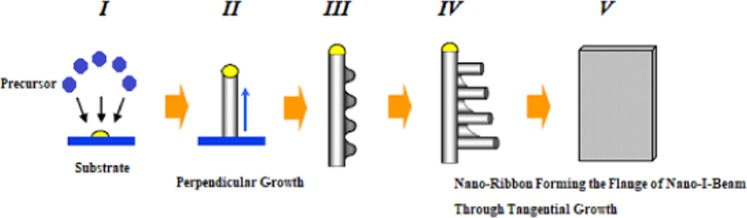
Evolution of the Nano-I-Beam Through Hybrid Growth Mode.

The evolution of the nano-I-beam is done through a hybrid growth mode. This hybrid growth mode is a combination of perpendicular growth mode of a nano-rod associated with a tangential growth mode of a ribbon of several nano-rods. The evolution of the nano-I-beam through the hybrid growth mode develops during five phases as shown in Fig. 2. These phases start with the interfacing and inter-phasing between the precursor and substrate, going through perpendicular growth of a nano-rod, associated with tangential growth of several nano-rods. These phases end with forming a nano-ribbon that constitutes the flange of the single-walled nano-I-beam primarily through tangential growth.

**Figure 3:**
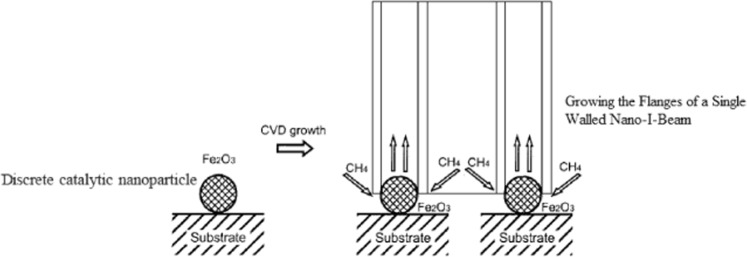
Synthesizing and Growing Discrete Single Walled Nano-I-Beam.

Discrete catalytic nanoparticles are favorably used for growth of single-walled nanotube (SWNTs) on substrates by chemical vapor deposition (CVD). The solvent and the structure and diameter of the catalytic nanoparticles allow the control of nanotube diameter, whilst the control of SWNT length is based on chirality^4^. By analogy, a discrete single walled nano-I-beam (SWNIB) can be synthesized and grown based on solid catalytic nanoparticles, as shown in Fig. 3.

**Figure 4:**
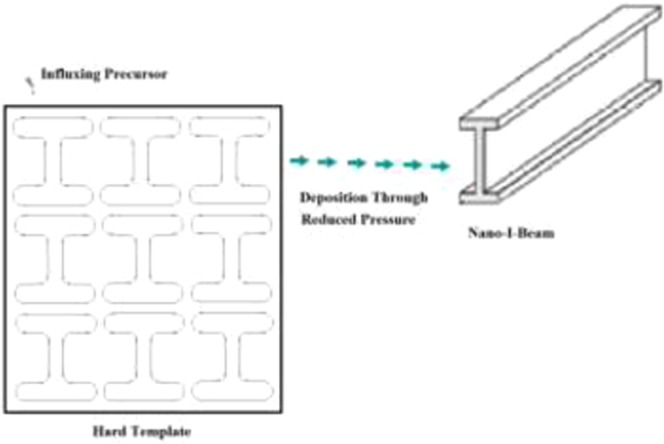
Synthesizing and Growing Ensemble of Single Walled Nano-I-Beams.

For larger scale synthesizing, an ensemble of SWNIBs can be synthesized and grown simultaneously together starting with in-fluxing precursor into a hard plate followed by deposition onto an I-shaped like template through reduced pressure and then removal of the template, as shown in Fig. 4. The solvent provides the reaction medium for the synthesizing of the precursor, whilst the precursor (e.g. nano-particle) then drives the growth of the nano-tube^5^. Having thus a pure solvent with dispersion energies per mole monomer of negative value ensures success of the synthesizing process^6^.

The Carbon nano-I-beam can also be synthesized through drawing/extrusion dies of I-like shape in order to produce bulk nano-I-beams of controlled electrical conductivity and controlled density. This can be done by analogy similar to the drawing of CNTs into bulk nano-wires proposed by Alvarenga *et al*.^7^. Aqueous ionic doping is recommended to be used as a doping agent and incorporated during the drawing/extrusion process as a lubricant. Carbon nano-I-beam is proposed to be partially drawn/extruded through a Tungsten Carbide die. The doping process typically leads to densification in terms of closer packed nano-I-beam and a reduced charge transfer barrier, resulting in enhanced bulk electrical conductivity. The Carbon nano-I-beam can also be synthesized as a single sheet of molecules that is synthesized and then rolled into a tube and then pressed by a nano- rectangular mandrel from both sides in a confined space of upper and lower flat surfaces. Most of the developed nano-structures such as nanotubes have open ends, while some of them have closed ends with the side of the tubes wrapped around the end to close it^5^.

Inorganic nanotubes are more recent than organic nanotubes and are morphologically similar to CNTs, but they are synthesized of metal oxides, group III-Nitrides, or other inorganic elements. Thus, inorganic nano-I-beams are promising for industrial applications such as: (i) Redox catalysts; (ii) Cathode materials for batteries; (iii) Fillers for composites with enhanced thermal, mechanical and electrical properties. Inorganic nano-I-beams are more meritorious than Carbon nano-I-beams in several senses including: (1) Easy synthesis, (2) High crystallinity though not perfect crystallinity, (3) Good uniformity and dispersion, (4) High impact-resistance, (5) Predefined electrical conductivity depending on the composition of the pre-synthesizing material and its morphology, (6) Good adhesion to a number of compounds. There are two factors that control the folding and growth behavior of the inorganic SWNTs and thus the uniformity of the outer dimensions of the evolving nano-structure: (I) Generating nuclei/ultrathin nanostructure in the proper solution, (II) The confining solvent/surfactant that interact^8^. These two controlling factors of growth behavior remain primarily true for the inorganic SWNIBs. This paves the way for investigating the evolution and kinetics of nano-I-beam.

## Evolution and Kinetics of Nano-I-Beams

**Figure 5:**
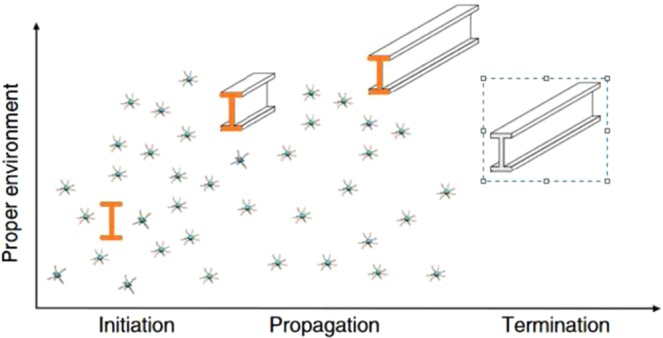
Kinetics of Formation of Single Walled Nano-I-Beam.

The kinetics of formation of single walled Carbon/inorganic nano-I-beam is based on three consecutive stages: initiation, propagation and termination as indicated in Fig. 5.

In case of aqueous solvent and surfactant, the chirality pull-out force is driven by: (i) the frictional sliding force caused by defects or preformed mechanical interlocking which often is insignificant and thus is neglected in modeling^9^, (ii) capillary interfacial force due to van der Waals and electro-static Coulombic interactions. The interfacial mechanism by which Carbon nano-I-beam and the surfactant interact depends on the Coulombic forces and the hydrophobic van der Waals interactions between the surfactant tail and the nano-I-beam walls. The surface chemical properties of the SWNIB such as the point of zero charge (PZC) depend on the pre-treatment (i.e. purification) of the surrounding medium, which can be acidic or basic. At low pH values, the surfactant is adsorbed on the growing wall of the nano-structure because the Coulombic forces are overcome by the hydrophobic van der Waals interactions^10^. This adsorption due to the hydrophobic force shapes the structure of the nano-I-beam. The catalytic nanoparticles gradually dissolve hydrothermally to generate free atoms that then drives the adsorption on the SWNIB. Only at pH values far from the PZC, the Coulombic forces become significant and the absorption becomes diminishing. The possible results of the evolution of nano-I-beams are thus now investigated.

## Single and Multi-Walled Nano- I-Beams

Single-walled carbon nanotubes are hollow cylinders that can grow millimeters long via Carbon incorporation at the interface with a catalyst. They display semiconducting or metallic characteristics, depending on their helicity, which is determined during their growth. The proposed nano-I-beam can be: (I) Solid single-walled Nano-I-Beam (the growth of which can be controlled through template); (II) Solid multi-walled Nano-I-Beam (the growth of which can be controlled through chirality and folding); (III) Hollow single-walled Nano-I-Beam (the growth of which can be controlled through template); (IV) or Hollow multi-walled Nano-I-Beam (the growth of which can be controlled through chirality and folding).

**Figure 6:**
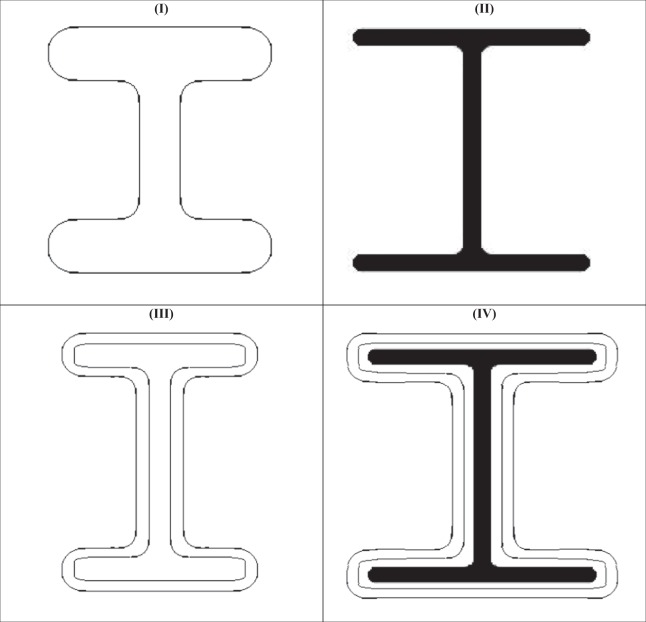
Single & Multi-Walled Nano-I-Beams. (**I**) Single Walled Hollow Nano-I-Beam Cross-Section; (**II**) Single Walled Solid Nano-I-Beam Cross-Section; (**III**) Multi-Walled Hollow Nano-I-Beam Cross-Section; (**IV**) Multi-Walled Solid Nano-I-Beam Cross-Section. These four figures indicate how flexibly promising the nano-I-beam can be in meeting the industrial needs in structural and electronics applications.

Single walled hollow nano-I-beam cross-section and single walled solid nano-I-beam cross-section can be developed as shown in Fig. 6. The innovation of the nano-I-beam can be extended into multi-walled hollow nano-I-beam cross-section, as shown in Fig. 6. Also, the innovation of the nano-I-beam can be extended into multi-walled solid nano-I-beam cross-section, as shown in Fig. 6.

These single-walled and multi-walled nano- I-beams exhibit exceptional structural strength that can efficiently serve the design for structural strength. Nano-I-beams also exhibit exceptional structural stiffness that can efficiently serve the design for structural stiffness.

## Structural Strength and Structural Stiffness of Nano-I-Beam

Papanikos *et al*.^11^, conducted finite element analysis to evaluate the geometrical characteristics and elastic properties of solid and hollow cylinders as equivalent to CNTs based on mechanics of materials. They concluded that the hollow cylinder shows equivalent beam to the carbon nanotube.

**Figure 7:**
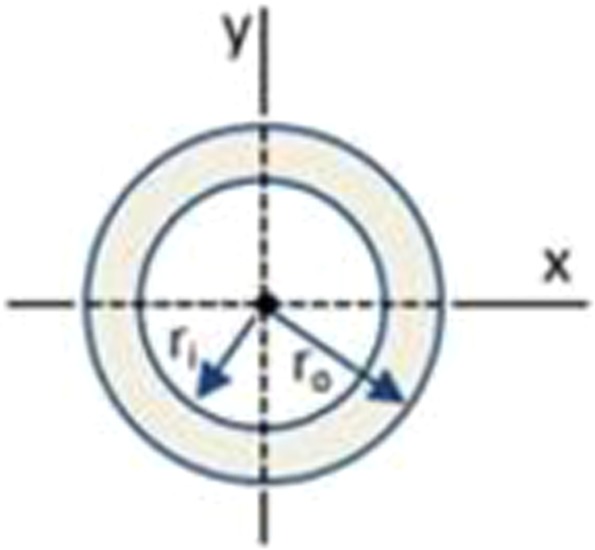
Circular tube cross-section.

**Figure 8:**
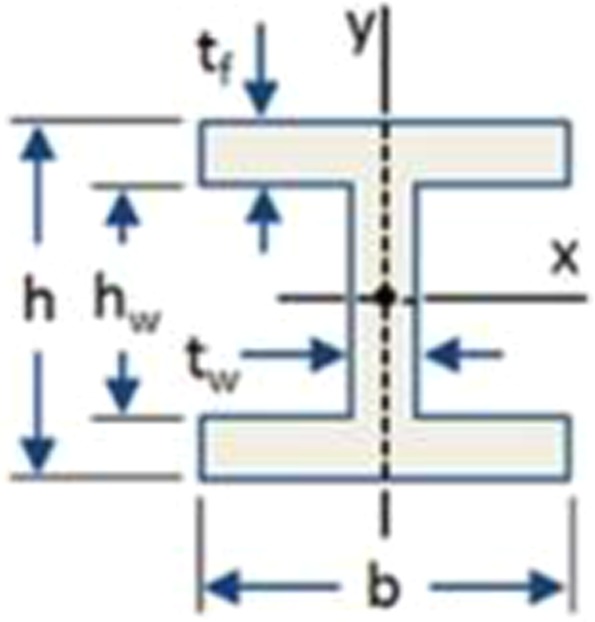
I-beam cross-section.

Let us first consider the design for structural strength. Bernoulli Euler theory is a key reference in the design of beams or flexural elements. In beam design the normal stress obtained from maximum moment (Mmax) is usually more significant than shear stress obtained from maximum shear (Vmax). Section modulus is a geometric property for a given cross-section used in the design of beams and flexural members for structural strength. Two cross-sections are considered herein: circular tube and I-beam, as shown in Figs. 7 and 8, respectively.

For ensuring accurate analysis, the same value of cross-sectional area should be maintained for the comparison of the circular tube cross-section and the I-like shaped cross-section. The cross-sectional area of the circular tube is analytically given as:

1 $${A}_{{\rm{C}}}=\pi ({{r}_{o}}^{2}-\,{{r}_{i}}^{2})$$

The cross-sectional area of the I-beam is analytically given as:

2 $${A}_{{\rm{I}}}={h}_{w}\,{t}_{w}+2\,b\,{t}_{f}$$

Typically the length of the nanotube ranges from 4 to 15 μm^12^. The diameter of the nanotube is often 15 nm^13^. Section modulus, *S*, indicates the value of the maximum moment that can be carried safely by the beam or flexural element, according to:

3 $$\sigma =\frac{M}{S}$$

The section modulus of the circular tube cross-section, *SC*, is analytically given as:

4 $${S}_{C}=\frac{\pi ({{r}_{o}}^{4}-\,{{r}_{i}}^{4})}{4\,{r}_{o}}$$

The section modulus of the I-beam cross-section, *SI*, is analytically given as:

5 $${S}_{I}=\frac{1}{6}\,[b\,{h}^{2}-\frac{{{h}_{w}}^{3}}{h}\,(b-{t}_{w})]$$

**Figure 9:**
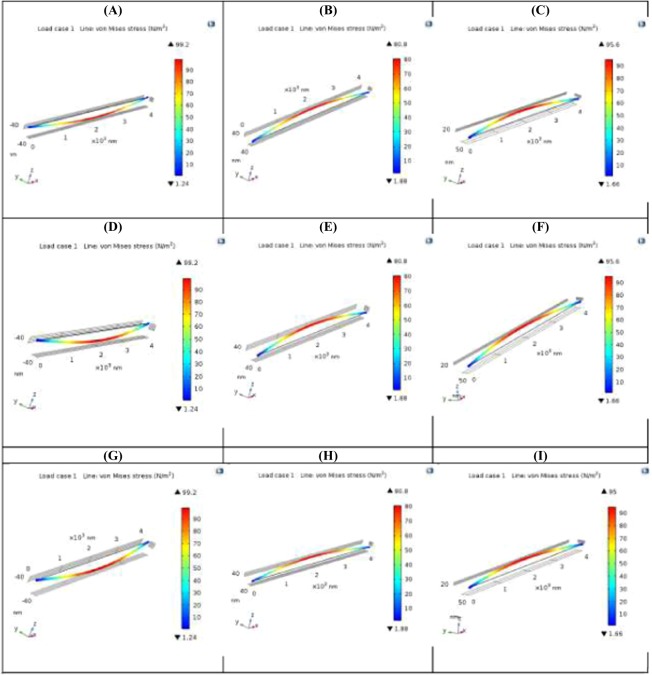
The Finite Element Modeling for the Two Cross-Sections on Structural Strength. (**A**) Maximum Induced Stress of Structural Steel Nano-Tube Section; (**B**) Maximum Induced Stress of Structural Steel Wide Flange Nano-I-Beam Section; (**C**) Maximum Induced Stress of Structural Steel Equal Flange & Web Nano-I-Beam Section; (**D**) Maximum Induced Stress of Graphite Sheet Nano-Tube Section; (**E**) Maximum Induced Stress of Graphite Sheet Wide Flange Nano-I-Beam Section; (**F**) Maximum Induced Stress of Graphite Sheet Equal Flange & Web Nano-I-Beam Section; (**G**) Maximum Induced Stress of C60 Fullerene Nano-Tube Section; (**H**) Maximum Induced Stress of C60 Fullerene Wide Flange Nano-I-Beam Section; (**I**) Maximum Induced Stress of C60 Fullerene Equal Flange & Web Nano-I-Beam Section. These nine figures indicate that both Wide Flange Nano-I-Beam and Equal Flange & Web Nano-I-beam provide less induced stress and thus longer service life than Nano-Tube. They also indicate that Wide Flange Nano-I-Beam provides less induced stress and thus longer service life than Equal Flange & Web Nano-I-beam.

For the design for structural strength, the two cross sections are thus compared herein using finite element analysis. The finite element modeling for the two cross-sections on structural strength is shown in Fig. 9. The computational results favor the Nano-I-Beam. The computational models of the structural strength of nano-I-beam based on the core computational engine of COMSOL Multi-physics are developed based on nano-scale mechanics of materials. The results of these models of the structural strength of nano-I-beam make sense in light of the provided analytical formulae of classical mechanics of materials. The provided analytical formulae of classical mechanics of materials Eqs. (3–5) are modified with factors in the core computational engine of COMSOL Multi-physics for taking into account the microscopic effects.

As to the design for structural stiffness, the moment of inertia is the second moment of area that indicates the ability of a beam or flexural member to resist bending. It determines the moment needed for desired bending about an axis. The moment of Inertia, *I*, indicates the value of the maximum deflection (*μ*) that can be experienced safely by the beam or flexural element, according to:

6 $$E\,I\,\frac{{d}^{2}\mu }{d{x}^{2}}=M(x)$$

The moment of inertia of the circular tube cross-section, *I*_*C*_, is analytically given as:

7 $${I}_{C}=\frac{\pi }{4}\,({{r}_{o}}^{4}-{{r}_{i}}^{4})$$

The moment of inertia of the I-beam cross-section, *I*_*I*_, is analytically given as:

8 $${I}_{I}=\frac{1}{12}\,(b\,{h}^{3}-b\,{{h}_{w}}^{3}+{t}_{w}\,{{h}_{w}}^{3})$$

**Figure 10:**
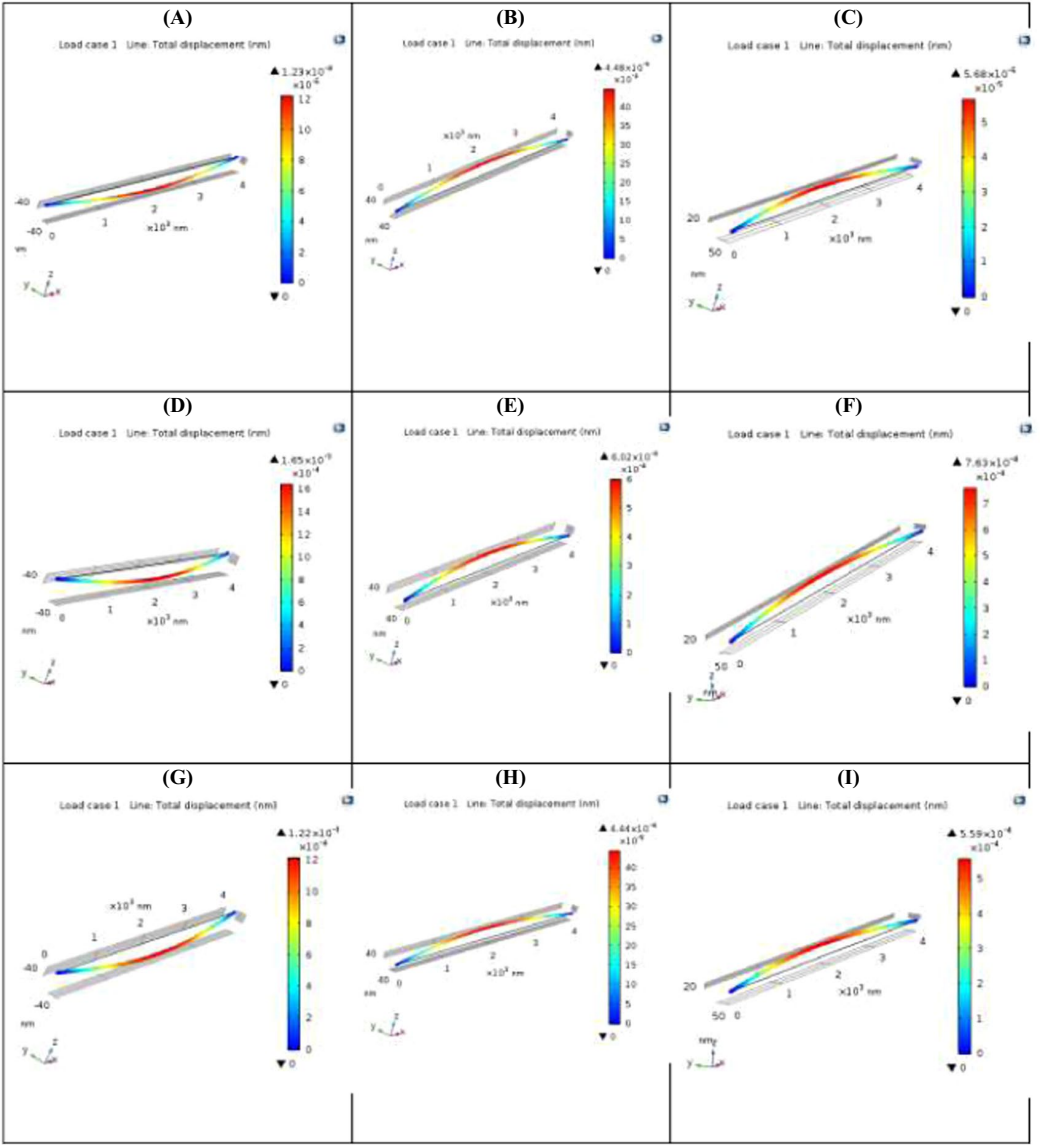
The Finite Element Modeling for the Two Cross-Sections on Structural Stiffness. (**A**) Total Displacement of Structural Steel Nano-Tube Section; (**B**) Total Displacement of Structural Steel Wide Flange Nano-I-Beam Section; (**C**) Total Displacement of Structural Steel Equal Flange & Web Nano-I-Beam Section; (**D**) Total Displacement of Graphite Sheet Nano-Tube Section; (**E**) Total Displacement of Graphite Sheet Wide Flange Nano-I-Beam Section; (**F**) Total Displacement of Graphite Sheet Equal Flange & Web Nano-I-Beam Section; (**G**) Total Displacement of C60 Fullerene Nano-Tube Section; (**H**) Total Displacement of C60 Fullerene Wide Flange Nano-I-Beam Section; (**I**) Total Displacement of C60 Fullerene Equal Flange & Web Nano-I-Beam Section. These nine figures indicate that both Wide Flange Nano-I-Beam and Equal Flange & Web Nano-I-beam provide higher structural stiffness than Nano-Tube. They also indicate that Wide Flange Nano-I-Beam provides higher structural stiffness than Equal Flange & Web Nano-I-beam.

Therefore, for the design for structural stiffness, the two cross sections are compared herein using finite element analysis. The finite element modeling for the two cross-sections on structural stiffness is shown in Fig. 10. The computational results favor the Nano-I-Beam. The computational models of the structural stiffness of nano-I-beam based on the core computational engine of COMSOL Multi-physics are developed based on nano-scale mechanics of materials. The results of these models of the structural stiffness of nano-I-beam make sense in light of the provided analytical formulae of classical mechanics of materials. The provided analytical formulae of classical mechanics of materials Eqs. (6–8) are modified with factors in the core computational engine of COMSOL Multi-physics for taking into account the microscopic effects.

In the interet of checking the ability of the structure to support load without undergoing unacceptable deformations, Euler’s critical load (*P*_*cr*_) formula can be applied with the conservative approach of the 1st mode cycle:

9 $${P}_{cr}=\frac{{\pi }^{2}\,E\,I}{{h}^{2}}$$

For the given E, I and h of standard aspect ratios, the Euler’s critical load (*P*_*cr*_) of Eq. (9) far exceeds the largest load applied within the elastic range onto the nano-I-beam (i.e. 6.9*10–12 N/m). Thus, the nano-I-beam is safe against unacceptable deformations. Having investigated the exceptional structural strength and structural stiffness of nano-I-beams, let us finally now thermodynamically model the kinetics and growth of nano-I-beams.

## Thermodynamic Model of the Kinetics and Growth of Nano-I-Beam and Nano-Tube

Modeling of the mechanisms underlying the growth of the SWNT and its selective synthesis is still a weak link in the development of the nano-structures research area^14^. For controlling such a selective synthesis, Magnin *et al*.^14^, developed a thermodynamic model that relates the tube-catalyst interfacial energies, temperature, and the resulting tube chirality. The present model herein shows the contribution of entropy of the nano-meter sized edge of the nano-tube to the total energy that shapes the temperature–based evolution of the nano-tube.

The electronic properties of the SWNTs and SWNIBs depend primarily on the way these nano-structures are rolled along their axis, i.e. chirality in stereoisomerism, characterized by two indices (*n*, *m*). The pair of indices (*n*, *m*) are two integers correspond to the number of unit vectors spatially in each unit (e.g. hexagonal) along the two directions in the organic honeycomb crystal lattice in a given cross-section of the tube/I-beam. When *m* = 0 the nanotube is called “Zigzag”, when *n* = m the nanotube is called “Armchair”, and all other configurations are called “Chiral”^15^. Controlling chirality during the synthesis of the tube/I-beam enables the nano-I-beam to have various industrial applications in sectors such as energy and electronics overcoming the limitations of Silicon.

Solid state catalysts have reported a chiral specific growth of SWNTs^16–18^. Puretzky *et al*.^19^, provided a model of the growth of SWNTs focusing on kinetics, but they neglected the role of the catalyst. Atomistic computer simulations emphasize on chemical accuracy^20,21^, but do not provide a mathematical thermodynamic model for understanding the process. Some researchers, such as^22^, attempted to present a model combining thermodynamic and kinetic aspects of the growth, but they overlooked the role of entropy, resulting in unrealistic results. Magnin *et al*.^14^ proposed thermodynamic modelling of the interface between the tube and the catalyst, but ignored the vibrational entropy contribution.

Here, an extended thermodynamic model of the interface between the tube and the catalyst is developed, to relate its properties to the resulting chiral distribution obtained during CVD synthesis experiments. Vapor-solid-solid chemical vapor deposition (CVD) synthesis is recommended to grow SWNTs leading to a (*n*, *m*) selectivity^17^. Growth can proceed through tangential or perpendicular modes^23^. The ways to control these modes have been proposed recently^24^. The present model is applicable to: (i) the perpendicular growth mode of the SWNT, (ii) the tangential growth mode of the SWNT where the lateral tube/catalyst interaction does not depend on (*n*, *m*), (iii) the perpendicular growth mode of the SWNIB, (iv) the tangential growth mode of the SWNIB where the lateral tube/catalyst interaction does not depend on (*n*, *m*).

The present thermodynamic system of growth of the SWNT/SWNIB is closed, so that the total number of atoms of the catalyst and tube is constant. By taking thermal equilibrium/in-equilibrium and irreversibility into account, the vibrational entropy contribution is introduced into the present model compensating for the atomic structure of the NP. Assuming that tribologically the catalyst appears as a smooth flat surface, the total energy of the growth system, *E* (*n*, *m*), at temperature *T* is:

10 $$E(\rho ,\theta ,\,z,t)={E}_{0}+{E}_{C}+{E}_{I}-T\,S$$

where:

*E*_*0*_ is the energy of the system in the standard state independent of the chirality indices (*n*, *m*);

*E*_*C*_ is the energy of the chirality curvature of the (*n*, *m*) SWNT/SWNIB;

*E*_*I*_ is the interface energy between the catalyst nanoparticle (NP), such as Carbide, and the SWNT/SWNIB.

For a given (*n*, *m*) of chirality in a micro-canonical ensemble in equilibrium so that energy is additive, the number of ways of putting (*n* − *m*) zigzag C atoms and *m* pairs of armchair atoms on *n* sites (degeneracy) are related through the principles of combinatorics. Magnin *et al*.^14^, thus thermodynamically modeled the entropy, S, following from Boltzmann’s definition of entropy as:

11 $$S\,(n,\,m)={k}_{B}\,ln\frac{n!}{m!\,(n-m)!}$$

Where:

*k*_*B*_ is Boltzmann constant.

The shell theory is often used for the analysis of CNTs. This is because the CNT is in fact a crystalline membrane with one atom thickness that can be considered as a homogeneous thin shell^25^. The Ritz method is often used in the dynamic analysis of CNTs based on the Shell Theory^25,26^. The Ritz method is related to the Hamilton’s principle in the following sense: According to the Hamilton’s principle, for a conservative structural system undergoing kinematically admissible growth/deformation, the equilibrium state minimizes the energy functional. The Hamilton’s principle relates the three elements of energy: the kinetic energy *T*, the strain energy *U*, and the potential energy *W*_*P*_.

**Figure 11:**
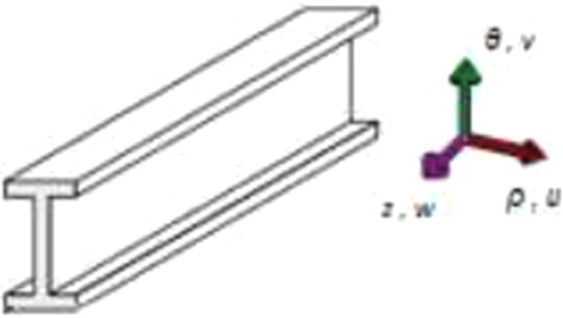
3-D Nano-I-Beam in Cylindrical Coordinate System.

For slashing the drawback of the model developed by Magnin *et al*.^14^, that makes their model of the total energy of the growth system a function of the chirality indices (*n*, *m*) rendering their model analytically unsolvable, the present research models the energy of the system as a function of the cylindrical coordinate system and temperature. Modeling the energy of the system as a function of the cylindrical coordinate system and temperature is in accord with the fact of having large number of degrees of freedom of this system. For the conservative system indicated in Fig. 11, the potential energy *W*_*P*_ of this conservative system is:

12 $${W}_{P}=q\,w(\rho ,\,\theta ,\,t)$$

Where: q is the attraction/repulsion force,

w (*ρ*, *θ*, *t*) is the out-of-plane displacement/growth

The strain energy *U* is formulated as the integration of the strain energy density:

13 $$U={\int }_{V}({\sigma }_{\rho }{{\epsilon }}_{\rho }+{\sigma }_{\theta }{{\epsilon }}_{\theta }+{\sigma }_{z}{{\epsilon }}_{z}+{\sigma }_{\rho \theta }{{\epsilon }}_{\rho \theta }+{\sigma }_{\rho z}{{\epsilon }}_{\rho z}+{\sigma }_{\theta z}{{\epsilon }}_{\theta z})dV$$

The free slippage of the molecular layers on top of each other is what influences the value of elastic strain energy, which is in turn crucial in ensuring success of the folding of the nano-structure. For kinetic energy, since observations are often made in a moving frame of reference, the time derivative of as observed in the fixed frame of reference (*ρ*, *θ*, *z*) is used. Thus, the kinetic energy *T* is formulated as:

14 $$T=\frac{1}{2}\,\gamma {\int }_{V}^{V}[{(\frac{\partial u}{\partial t})}^{2}+{(\frac{\partial v}{\partial t})}^{2}+{(\frac{\partial w}{\partial t})}^{2}]\,dV$$

where:

γ is the density.

*u* (*ρ*, *θ*, *t*) and *v* (*ρ*, *θ*, *t*) are the in-plane displacement.

Considering the energy system as a collection of infinitesimal oscillators, for the present conservative system, the Hamilton’s principle relates the elements of the energy of the chirality curvature of the SWNT/SWNIB, *EC*, following from the Lagrangian as:

15 $${E}_{C}=T-U-{W}_{P}$$

For the energy of the chirality curvature of the SWNT/SWNIB, EC, in this conservative system the coordinates (*θ*, *z*) become variable while the third coordinate *ρ* becomes constant. Thus:

16 $${E}_{C}={\int }_{V}[\frac{\gamma {(\frac{\partial v}{\partial t})}^{2}+\gamma {(\frac{\partial w}{\partial t})}^{2}}{2\,}-({\sigma }_{\rho }{{\epsilon }}_{\rho }+{\sigma }_{\theta }{{\epsilon }}_{\theta }+{\sigma }_{z}{{\epsilon }}_{z}+{\sigma }_{\rho \theta }{{\epsilon }}_{\rho \theta }+{\sigma }_{\rho z}{{\epsilon }}_{\rho z}+{\sigma }_{\theta z}{{\epsilon }}_{\theta z})]dV-q\,w(\rho ,\,\theta ,\,t)$$

Also, for this conservative system, the Hamilton’s principle relates the elements of the energy of the interface energy between the catalyst nanoparticle and the SWNT/SWNIB, *E*_*I*_, where the coordinate (*θ*) becomes variable and the coordinates (*ρ*, *z*) become constant. Thus, following from the Lagrangian and by analogy to Eq. (15), the interface energy between the catalyst nanoparticle and the SWNT/SWNIB, *E*_*I*_, in this conservative system is:

17 $${E}_{I}={\int }_{V}[\frac{\gamma \,{(\frac{\partial v}{\partial t})}^{2}}{2}-({\sigma }_{\rho }{{\epsilon }}_{\rho }+{\sigma }_{\theta }{{\epsilon }}_{\theta }+{\sigma }_{z}{{\epsilon }}_{z}+{\sigma }_{\rho \theta }{{\epsilon }}_{\rho \theta }+{\sigma }_{\rho z}{{\epsilon }}_{\rho z}+{\sigma }_{\theta z}{{\epsilon }}_{\theta z})]dV-q\,w(\rho ,\,\theta ,\,t)$$

The positive term of the interface energy represents the energy release due to cutting the bonds of the tube at the edge. The negative term of the interface energy represents the adhesion energy needed to be got for reconnecting a cut edge of the tube and the nano-particle (NP). For maintaining a driving potential of formation of the SWNT/SWNIB, the positive term of the interface energy has to be larger than the negative term thereof. Larger diameter tubes are obtained for small values of interface energy because the entropy cannot then counterbalance the energy cost of the interface.

Also, for this conservative system, the Hamilton’s principle relates the elements of the energy of the system in the standard state, *E*_*0*_, where the coordinates (*θ*, *z*) = (0, 0) and the third coordinate ρ becomes constant. Thus, following from the Lagrangian and by analogy to Eq. (15), the energy of the system in the standard state is:

18 $${E}_{0}={\int }_{C}[-({\sigma }_{\rho }{{\epsilon }}_{\rho }+{\sigma }_{\rho \theta }{{\epsilon }}_{\rho \theta }+{\sigma }_{\rho z}{{\epsilon }}_{\rho z})]\,dV-q\,w(\rho ,\,\theta ,\,t)$$

Therefore, the total energy of the growth system, *E* (*n*, *m*), at temperature T becomes:

19 $$\begin{array}{ccc}{\rm{E}}({\rm{\rho }},{\rm{\theta }},\,{\rm{z}},{\rm{t}}) & = & {\int }_{V}[\gamma \,{(\frac{\partial v}{\partial t})}^{2}+\frac{\gamma \,{(\frac{\partial w}{\partial t})}^{2}}{2\,}\\  &  & -2\,(2\,{\sigma }_{\rho }{{\epsilon }}_{\rho }+{\sigma }_{\theta }{{\epsilon }}_{\theta }+{\sigma }_{z}{{\epsilon }}_{z}+2\,{\sigma }_{\rho \theta }{{\epsilon }}_{\rho \theta }+2\,{\sigma }_{\rho z}{{\epsilon }}_{\rho z}+{\sigma }_{\theta z}{{\epsilon }}_{\theta z})]dV\\  &  & -3\,q\,w(\rho ,\,\theta ,\,t)-T\,{k}_{B}\,ln\frac{n!}{m!\,(n-m)!}\end{array}$$

The interfacial energy (*E*_*I*_) is expected to be low in comparison with the other two elements of the total energy (*E*_*C*_ and *E*_*0*_) due to the relaxation (i.e. shortening) of the C-C bonds of the armchair interfacing edge for the perpendicular growth that thus results in stabilizing the interface of the tube. This is further supported by the fact that the adhesion energies of SWNT/SWNIB of armchair and zigzag terminations in contact with typical catalysts on icosahedral clusters of substrate of various metals, lie between 0.0 and 0.5 eV/bond^14^. The energy terms *E*_*0*_, *E*_*C*_, and *E*_*I*_ in Eq. (19) can be evaluated using the Density Functional Theory (DFT) calculations that are given in^14,27–29^.

The driving force for the growth of the Carbon SWNT/SWNIB stems from the abundant reactive atoms on the periphery of the nanostructure. These rim atoms are only twofold bonded rather than being threefold (sp^2^) bonded as in the bulk. Thus, in the case of organic SWNTs/SWNIBs, the threefold (sp^2^) bonded carbon atoms of the bulk of the carbon SWNTs/SWNIBs render these structures energetically stable. However, the deviation from planarity due to the rolled form induces stress into their structure explaining many of their chemical and physical properties. In the case of inorganic SWNT/SWNIB, the bending energy of several isotropic (3-D) inorganic materials is exceedingly higher than that of 2-D compounds due to the dangling bonds, which makes their folding into stably crystalline nanotubes unlikely. Thus, in such a case, synthesis of semi-crystalline inorganic SWNTs/SWNIBs is proposed through template-based growth.

The present model may result in some overestimation/underestimation because of the influence of side parameters not explicitly incorporated into the model, such as the variability in the catalyst size, chemical composition, and atomic structure. Such variability can result in intrinsic disorder at the edge of the SWNT/SWNIB. The present model would help in relating the properties of the SWNT/SWNIB to the chiral distribution obtained during the CVD synthesis.

## Discussion

Both energy and matter have fundamental building blocks: quanta and atoms, respectively. The present research addresses both of these building blocks. An explanation of the reduced induced stress in the nano-I-beam is the improved flow stress throughout the cross-section out-performing the nano-tube. Carbon/Inorganic nano-I-beams have cost-effectively several potential benefits in industrial applications such as nano-heat engines, nano-actuators, and nano-sensors that address both energy and matter.

The stability of flange and web becomes questionable if the load is dynamic, or the dimensional aspect ratios are not standard, or the load exceeds the critical Euler’s critical load formula (i.e. Buckling instability criterion). The investigated load case is quasi-static. The investigated dimensional aspect ratios are standard. The load does not exceed the Euler’s critical load. Thus, the stability of flange and web of the nano-I-beam would not compromise the results herein. However, from conservative perspective, for the computational models developed herein a couple of assumptions are made herein on large deformations and the investigated load case, as mentioned in the section of “Materials and Methods”.

The hybrid semi-conduction feature in the flange and web of the nano-I-beam is promising for nano-devices. This one-dimensional nanomaterial can thus help shape the next-generation electronic systems having small size, faster transport speed, higher efficiency, and less energy consumption. The unique geometrical characteristics of the nano-I-beam exhibit exceptional acoustic and thermal properties of Carbon nano-I-beam. These acoustic and thermal properties are highly anisotropic since phonons propagate very quickly along the tightly-bound planes of the flange of the nano-I-beam, but are slower to travel from one plane to another in the web of the nano-I-beam. This acoustic and thermal anisotropy can be explained in light of the fact that the smaller the confining dimensions of the nanostructure (i.e. the confinement width of the quantum well), the wider is the separation between the energy levels, leading to a unique spectrum of discreet/discrete energies.

Amongst the promising industrial applications of the present research is to apply it onto Piezoelectric materials which are ideal for various types of sensors. Virtually all piezoelectric materials are ceramics, which are far from ideal for applications requiring exceptional mechanical properties^30^. Carbon/inorganic nano-I-beams suit a molecular solid-solution series that allows for compositional optimization of the piezoelectric properties termed morpho-tropic phase boundaries (MPBs).

Electro-mechanical actuators based on sheets of Carbon SWNTs were shown to generate higher strains than high-modulus ferroelectrics. Actuators using optimized nanotube sheets provide substantially higher work densities per cycle than any previously known technology^31^. Thus, hybrid organic/inorganic nano-I-beams have potential industrial application in electro-mechanical actuators providing higher structural stiffness and less induced stress and thus longer service life and higher work densities per cycle than high-modulus ferroelectrics.

Bulk hybrid organic/inorganic nano-I-beams particulate/laminated composite have the industrial potential to make the fuselage and skin of jet aircrafts and gliders of nano-composite honey comb structure that acts as a super-capacitor through a thin-film and/or large-area coatings that store solar energy while simultaneously provide structural stiffness and strength. Bulk hybrid organic/inorganic nano-I-beams particulate/laminated composite yarns and sheets can be made of Silicon Germanium (SiGe) Oxides and be incorporated in the fuselage and skin of jet aircrafts adjacent to the burner of the jet engine acting as a thermoelectric material that generates power directly from heat by converting temperature differences into electric voltage. This material has both high electrical conductivity (*σ*) and low thermal conductivity (*κ*) to be a good thermoelectric material. Having low thermal conductivity ensures that when one side is made hot, the other side stays cold, which helps in generating a large electric potential while in a temperature gradient. Such a thermoelectric circuit can be constructed based on materials of different Seebeck coefficient (p-doped and n-doped semiconductors), configured as a thermoelectric generator. The power factor is directly proportional with the square of Seebeck coefficient. The thermal conductivity of semiconductors can be lowered without affecting their high electrical properties using this nanotechnology in making this bulk semiconductor material.

Other researchers innovated materials that can be also integrated with this proposed innovation. Lin and Zhao^32^ modeled the building of van der Waals (vdW) heterostructures as the construction of a variety of layered structures, i.e. 2D crystals, resembling atomic-scale Lego blocks. The nano-I-beam can be integratively grown based on the vdW heterostructures. Elmoselhy^33,34^ proposed the alloying of a heterogeneous composite ferromagnetic metallic glass matrix, such as steel matrix, with nano-meter sized elements and the mass production of the alloying of this matrix with nano-meter sized elements in order to produce bulk metallic glasses for industrial sectors such as automotive. The innovation of heterogeneous glass (i.e. crystalline glass) for 3-D material (i.e. bulk material) has been proved experimentally valid in 2014^35^. Thus, the proposed nano-I-beam can be integrated into such a composite matrix for improved properties.

## Conclusion

The present article has presented the Hybrid Organic/Inorganic Nano-I-Beam for structural nano-mechanics. It brings about the following contributions:

(I) Hybrid Organic/Inorganic Nano-I-Beam is introduced for improved structural nano-mechanics;
(II) Both Wide Flange Nano-I-Beam and Equal Flange & Web Nano-I-beam provide higher structural stiffness and less induced stress and thus longer service life than Nano-Tube;
(III) Wide Flange Nano-I-Beam provides higher structural stiffness and less induced stress and thus longer service life than Equal Flange & Web Nano-I-beam;
(IV) A thermodynamic model of the growth of nano-tubes accounting for vibrational entropy.

## Materials and Methods

This research work is quantitative and is based on a theoretical approach employing exploratory and computational techniques. Analytical models are employed in this research for showing rationality of the computational models. From conservative perspective, for the computational models developed herein the assumptions in this research include: (i) Large deformations are usually accompanied in nanostructures, so that only small deformation is considered herein; (ii) The investigated load case is quasi-static.

The finite element modeling and analysis have been adopted in this research. COMSOL Multi-Physics modeler has been used in both the Nanotube and nano-I-Beam models. The majority of the previous studies on Carbon nanotubes modeled the mechanical boundary conditions of the Carbon nanotubes with all edges simply supported for which an exact analytical solution exists^25^. Therefore, the mechanical boundary conditions of the Nanotube and Nano-I-Beam modeled in the present paper are with all edges simply supported.

In the finite element models, the standard aspect ratios of the standard sections have been employed: (i) For wide flange I-beam (i.e. w-beam), the European Standard Beams HE 120 A; (ii) For the equal/near-equal flange & web I-beam (i.e. H-beam), the American Standard Beams AISC W5x16. A nanotube is often 15 nanometer wide (i.e. *b* = 15 nm)^13^. Thus, the outer diameter of the circular tube *r*_*o*_ = 7.5 nm. Therefore, the cross-sectional area of the nano-I-beam can be readily calculated from its analytical formula provided above: *A*_*I*_ = 52.1 nm^2^. By applying the equally corresponding cross-sectional area on the nanotube, the inner radius of the nanotube can be readily calculated from the analytical formula of the cross-sectional area of the circular tube to be *r*_*i*_ = 6.3 nm. The length of the nanotube is typically 4 micro meter = 4000 nm^13^, and correspondingly so be the length of the nano-I-beam. The structural alloy steel is ASTM grade A723/A723M Class 1 (Yield Strength of 690 MPa). Thus, the largest load applied within the elastic range onto the simply supported beam in the three coordinate directions is 6.9*10^−12^ N/m.

The Graphite Sheet used in the finite element modeling is characterized in terms of the following properties^36^: Young’s modulus = 15.8 GPa; Poisson’s ratio = 0.2. The C60 Fullerene used in the finite element modeling is characterized in terms of the following properties^37^: Young’s modulus = 1.4 GPa; Poisson’s ratio = 0.34.

## Data Availability

All data is available in the main text or the supplementary materials. All data, code, and materials used in the analysis is available upon request to any researcher for purposes of reproducing or extending the analysis.
